# Mechanotransductive Differentiation of Hair Follicle Stem Cells Derived from Aged Eyelid Skin into Corneal Endothelial-Like Cells

**DOI:** 10.1007/s12015-021-10249-0

**Published:** 2021-09-13

**Authors:** Christian Olszewski, Jessika Maassen, Rebecca Guenther, Claudia Skazik-Voogt, Angela Gutermuth

**Affiliations:** Fraunhofer Institute for Production Technology, Steinbachstraße 17, 52074 Aachen, Germany

**Keywords:** Neural crest stem cells, Hair follicle stem cells, Corneal endothelial-like cells, Mechanotransduction

## Abstract

Corneal endothelial insufficiency is one of the leading causes of blindness. The main contemporary treatment for corneal blindness is endothelial keratoplasty, which, however, is unsatisfactory as a medical therapy due to the lack of donor corneas and graft rejection. Therefore, autologous stem cell-based corneal endothelial tissue substitutes may be a promising alternative to conventional grafts in the future. To address the age of most patients suffering from corneal endothelial deficiencies, we investigated the presence and potential of hair-derived stem cells from older tissue donors. Our studies revealed the presence of pluripotency- and neural crest-associated markers in tissue sections from blepharoplasty patients aged 50 to 80 years. In vitro outgrowths from eyelid hair follicles on collagen-coated tissue culture plates revealed a weak decrease in stem-cell potency. In contrast, cells within the spheres that spontaneously formed from the adherent cell layer retained full stem-cell potency and could be differentiated into cells of the ecto- meso and endodermal lineages. Although these highly potent hair follicle derived stem cells (HFSC) were only very slightly expandable, they were able to recognize the biomimicry of the Descemet’s-like topography and differentiate into corneal endothelial-like cells. In conclusion, HFSCs derived from epidermal skin of eyelid biopsies are a promising cell source to provide autologous corneal endothelial replacement for any age group of patients.

**Graphical Abstract:**

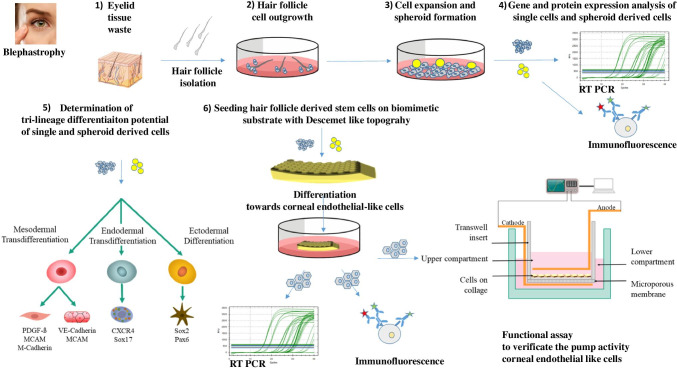

**Supplementary Information:**

The online version contains supplementary material available at 10.1007/s12015-021-10249-0.

## Introduction

The corneal endothelium covers the posterior surface of the cornea and is essential for clear vision as it maintains corneal hydration, thickness and transparency [1].

Endothelial insufficiency may occur in cases of corneal injury or inherited or acquired endotheliopathies, postsurgical endothelial decompensation [2, 3], glaucoma-associated increased intraocular pressure [4, 5], pharmacologically induced edema [6], but can also be caused by wearing contact lenses [7]. This may result in corneal opacity or even blindness due to the insufficient regenerative capacity of the corneal endothelial tissue. Although corneal endothelial progenitor cells have been detected in adult tissue, they make a limited contribution to replacing lost cells to restore the cell density necessary for a fully functional corneal endothelium [8]. To date, penetrating keratoplasty, transplantation of a full-thickness donor cornea, or endothelial lamellar keratoplasty, as in Descemet’s Stripping (Automated) Endothelial Keratoplasty (DSAEK) and Descemet Membrane Endothelial Keratoplasty (DMEK), are the only therapeutic options in case corneal endothelial decompensation occurs. Both penetrating and lamellar keratoplasty require the use of a donor cornea with at least 2,000 endothelial cells/mm^2^ to compensate for increased postsurgical cell loss.

Worldwide, corneal blindness affects approx. 2 million people annually, which could be cured by a corneal graft [9]. Donor corneas can be stored in organ culture under physiological conditions for up to four weeks. However, approximately 20% of all acquired donor corneas are unsuitable for keratoplasty due to an insufficient endothelium, caused e.g., by cell death during organ culture. Processing corneas from older donors cannot overcome this lack of donor tissue because the proportion of qualitatively adequate corneas decreases with donor age [10–13].

Not only is there a worldwide shortage of donor corneas, but the transplantation of allogenic tissue substitute is also problematic, associated with lifelong administration of immunosuppressant agents to avoid graft rejection or graft failure [14, 15].

Thus, there is great demand for alternative therapies to help patients with corneal endothelial deficiencies [16]. The idea of substituting the dysfunctional corneal endothelial layer with a tissue-engineered, autologous, stem-cell-based, corneal endothelial layer seems promising. Our research group has previously shown that Wharton’s Jelly-derived endothelial precursor cells (EPCs) are able to transdifferentiate into corneal endothelial-like cells upon mechanotransductive induction on a biomaterial with a Descemet’s membrane like topography (DLT) [17]. Considering that stem-cell-based grafts from umbilical cord tissue would be allogenic in origin, we aim to advance our approach by developing a corresponding autograft.

The most promising strategy is to select a stem-cell source that gives rise to the target cell line, in our case corneal endothelium cells. Extensive studies on avian eyes have demonstrated that during embryonic ocular eye development, a loose array of neuro-ectoderm-derived cranial neural crest cells, referred to as periocular mesenchyme (POM), migrate into the optic cup and give rise to various structures of the eye, including the corneal endothelium [18, 19]. Studies by Hara et al. [20] suggest that remnants of POMS are still present between adult corneal endothelial cells. They identified corneal endothelial progenitor cells expressing typical markers of the neural crest and periocular mesenchyme such as p75 neurotrophin receptor, SOX9 and FOXC2. However, in the context of tissue engineering, this cell source is only suitable for the generation of allogenic stem-cell-based transplants, as the isolation of the CEPs requires the digestion of the whole corneal endothelial tissue. Another obvious possibility would be to utilize cranial neural crest-derived stem cells from the immediate vicinity, which share a similar developmental history. Recently, Inagaki et al. successfully differentiated eyelid skin-derived neural crest precursor cells into corneal endothelial-like cells using biochemical differentiation factors and, thus, demonstrated the still existing potency of stem cells from elderly patients [21].

The objective of this study was to differentiate eyelid-derived epidermal hair follicle neural crest stem cells (HF-NCSCs) by biomimetic induction using our well established tool that simulates the Descemet’s environment [22]. After the high potency of HFSCs was first demonstrated, the cells were seeded onto a DLT-imprinted membrane and differentiated into corneal endothelial-like cells under FCS-free cultivation conditions and without the use of biochemical differentiation factors.

## Materials and Methods

### Histology

Histology investigations were performed according to the protocol of Fisher et al. [18]. Briefly, tissue slides were generated from eye lid tissue from blephastrophy patients and histologically analyzed and were therefore stained with hematoxylin and eosin as followed. For a hematoxylin and eosin stain, tissue sections were deparaffinized through xylene incubation three times for five minutes. Rehydration was performed with a descending series of alcohol, which consisted of two incubation steps in absolute ethanol (> 99.9%), two steps in 95% ethanol and one in 70% ethanol for five minutes each. Afterwards, sections were briefly washed in distilled water, stained in Harris hematoxylin solution for eight minutes and washed again in running tap water for five minutes. Bluing occurred in 0.2% ammonia water for one minute and thereupon sections were washed in running tap water for five minutes. The sections were rinsed and dipped ten times in 95% ethanol. Counterstaining with eosin solution was carried out for 60 s and sections dehydrated in an ascending series of alcohol: twice in 95% ethanol, twice in absolute ethanol (> 99.9%) and thrice in xylene for five minutes each. Sections were mounted with xylene-based Roti® HistoKitt II (Karlsruhe, Germany) mounting medium.

For immunostaining, blocking of unspecific antibody binding was performed with 10% normal goat serum with 2% bovine serum albumin (BSA) in PBS + / + for 60 min. Cells or tissue sections were then incubated with the primary antibody for membranous proteins with a titrated antibody dilution for an hour at room temperature or overnight at 4 °C in a moistly chamber. After a washing with PBS + / + with 0.1% BSA, cells or sections were perme-abilized 0.1% Triton X-100 for 10 min at room temperature and subse-quently incubated with the primary antibody staining for nuclear proteins or the secondary antibody for 30–60 min respectively in a moistly chamber at room temperature. Lastly after washing, 1:10,000 diluted DAPI was ap-plied for 10 min in a moistly chamber at room temperature for the visuali-zation of nuclei. Cells were mounted using Shandon™ Immu-Mount™ (Thermo Scientific, USA) onto a slide and tissue sections were mounted with a cover glass. Used primary antibodies were: CD34-PE 1:50; CD144 1:11, CXCR4-PE 1:50, Pax6-PE 1:11, PDGFR beta-VioBright FITC 1:10, Sox17-Vio515 1:50, Tra-1–60-PE 1:50, all Miltenyi Biotec, ATP1A1 1:100, beta-tubulin III 1:500, Connexin-43 1:300, Nestin 1:200, SSEA-4 1:200, Sox2-Alexa Fluor 488 1:100, Synaptophysin 1:500, ZO-1 1:500, all Thermo Fisher Scientific, Collagen Type VIII Alpha 2, 1:50, Novus Biologicals and PITX-2 1:500, abnova. Used secondary antibodies were Goat anti-Mouse IgG DyLight 594 1:250, Goat anti-Mouse IgG DyLight 650, 1:250 and Goat anti-Rabbit IgG DyLight 488 1:250, all Thermo Fisher Scientific.

### Cell Isolation

Donors underwent blepharoplasty surgery in order to get their blepharochalasis removed. Eyelid samples were obtained from adult, mostly female (female *n* = 10, male *n* = 3) patients with a mean age of 53.5 ± 7.1 years, which had given informed consent. Working in sterile conditions, tissue was cleaned in 70% ethanol and then rinsed with DPBS- (Life Technologies, Darmstadt, Germany). Any excess adipose tissue and blood coagulations were removed. Subsequently, the tissue samples were subjected to enzymatic digestion in 10 mg/ml Dispase II (Life Technologies, Darmstadt, Germany) overnight at 4 °C. Individual hairs were pulled off the skin by grasping the hair shaft directly above the epidermis with medical forceps and pulling firmly on it. Hair follicles were incubated in 0.05% Trypsin/EDTA (Life Technologies, Darmstadt, Germany) for 20 min at room temperature with periodic shaking. Trypsinization process was inhibited by addition of 10% FBS containing media. The trypsinized follicular epithelium was filtered through a 40 µM cell strainer. Cells were centrifuged with 300 RCF for 5 min, supernatant was discarded and cell were resuspended in 2 mL hair follicle stem-cell proliferation media consisting of DMEM/F-12 (Life Technologies, Darmstadt, Germany), 1% Pen/Strep (Life Technologies, Darmstadt, Germany), 1% Amphotericin B (Life Technologies, Darmstadt, Germany), 0.1% Ciprofloxacin (SERVA, Heidelberg, Germany), 1% N-2 (Life Technologies, Darmstadt, Germany), 2% B27 (Life Technologies, Darmstadt, Germany), 50 µM 2-Mercaptoethanol (Life Technologies, Darmstadt), 20 ng/mL bFGF (STEMCELL Technologies, Grenoble, France), 20 ng/mL IGF-1 (Life Technologies, Darmstadt) and 20 ng/mL EGF (Sigma-Aldrich, St. Louis, USA). in a 6-well plate coated with collagen I from rat tail (Life Technologies, Darmstadt, Germany) with a concentration of 50 µg/ml.

### Flow Cytometry

Cells were detached by Accutase® solution (Sigma Aldrich, USA), according to the manufacturer’s instructions. The single cell suspension was incubated in 4% formaldehyde for 10 min at 4 °C in the dark. After washing with DPBS + , cells intended for nuclear staining were incubated with 0.1% Triton-X-100 for 10 min in the dark. After another washing step, cells were incubated with blocking solution (consisting of 2% BSA in DPBS + with 10% Normal Goat Serum). Subsequently, cells were incubated with a directly-labeled primary antibody according to the manufacturer’s data sheet. After centrifugation, cells were resuspended in 600 µL FACS buffer consisting of DPBS + with 0.5% FBS and 2 mM EDTA). Cells were analyzed for CD271 (REA human 1:50) Nanog (REA human 1:50) and Sox2 (REA human 1:50) antigen expression as well as for unspecific antibody binding using according REA Isotype controls. All antibodies were costumized from Miltenyi Biotec, Bergisch Gladbach. The measurement was proceeded with BD FACSCantoII at the Flow Cytometry Facility of the IZKF, University Hospital RWTH Aachen.

### Real Time PCR

RNA isolation was performed by QIAGEN’s RNeasy Micro Kit (QIAGEN, Venlo, Netherlands). cDNA synthesis was conducted with 500 ng of total RNA using Bio-Rad’s iScript™ Select cDNA Synthesis Kit (Bio-Rad, Hercules, USA). If the RNA concentration was too low to employ 500 ng in cDNA synthesis, cDNA was synthesized from total RNA and then pre-amplified using Bio-Rad’s SsoAdvanced™ PrimePCR™ PreAmp Kit (Bio-Rad, Hercules, USA). Real-time polymerase chain reaction was performed with Bio-Rad’s SsoAdvanced™ PrimePCR™ SYBR Green Supermix (Bio-Rad, Hercules, USA) utilizing the following primer for housekeeping genes B2M and GAPDH with the assay IDs qHsaCID0015347 and qHsaCED0038674 and for regulated genes *APT1A1*, *PITX2*, *COL8A1*, TJP1 with assay IDs qHsaCED0046076, qHsaCED0046172, qHsaCID0036608 and qHsaCID0018062, respectively. Stem-cell plasticity was investigated by RT-PCR for the following genes: *c-MYC, KLF44, NANOG, POU5F1* and *SOX2.* X-fold expression change of adherent cells and spheres was investigated and normalized to gene expression of the Jurkat J6 cell line. Corneal endothelial gene RT-PCR was analyzed for the relative expression change compared to tissue culture plate control RNA samples for the expression of *ATP1A1, PITX2, COL8A2* and *TJP1.*

### (Trans-)Differentiation Capability Assessment

The differentiation and transdifferentiation capability were assessed by Miltenyi Biotec’s StemMACS™ Trilineage Differentiation Kit, human (Miltenyi Biotec, Bergisch Gladbach, Germany) as described by the manufacturer’s data sheet. Briefly, cells and spheres were cultivated in Miltenyi Biotec’s StemMACS™ iPS-Brew XF (Miltenyi Biotec, Bergisch Gladbach, Germany) medium. Subsequently, cells were re-seeded on Corning® Matrigel® hESC-qualified Matrix (Corning Life Sciences, Amsterdam, Netherlands) coated coverslips with 80,000 cells for mesodermal differentiation in StemMACS™ iPS-Brew XF with 10 µM StemMACS™ Y27632 (Miltenyi Biotec, Bergisch Gladbach, Germany), 130,000 cells for endodermal differentiation in StemMACS™ iPS-Brew XF with 10 µM StemMACS™ Y27632 (Miltenyi Biotec, Bergisch Gladbach, Germany) and 100,000 cells for ectodermal differentiation in StemMACS™ Trilineage EctoDiff Medium (Miltenyi Biotec, Bergisch Gladbach, Germany) in a 24-well plate. For mesodermal differentiation the medium was exchanged to StemMACS™ Trilineage MesoDiff Medium I (Miltenyi Biotec, Bergisch Gladbach, Germany) one day after seeding and subsequently to StemMACS™ Trilineage MesoDiff Medium II (Miltenyi Biotec, Bergisch Gladbach, Germany) four days after initial plating. For endodermal differentiation StemMACS™ Trilineage EndoDiff Medium (Miltenyi Biotec, Bergisch Gladbach, Germany) was used on the second day after the initial plating. Media change was performed as instructed by the data sheet.

### Differentiation into Corneal-Endothelial-Like Cells

Spheres formed from adherent colony-forming cells from passage 0 and 1 were harvested by mechanical force. Spheres acquired by this method were then seeded on a collagen I (Roche, Basel, Switzerland) membrane with a DLT as previously described in our publications. Cells were cultivated for 24 days in Endothelial Serum-Free medium (ESFM, Life Technologies, Darmstadt, Germany) supplemented with 20 ng/ml of bFGF (STEMCELL Technologies, Grenoble, France), 20 ng/ml of EGF, 1% Pen/Strep and 0.1% ciprofloxacin. Medium was changed every two to three days until a confluent layer of cells was visible.

### Immunofluorescence Measurement

Cells were seeded out on collagen I coated glass coverslips and were used for staining immediately after fixation with 4% paraformaldehyde for ten minutes in the refrigerator (4 °C).

Blocking of unspecific staining was performed with 10% normal serum from the host of the fluorescent secondary antibody with 2% bovine serum albumin (BSA) in PBS + / + for 30 to 60 min. Cells were then incubated with the primary antibody with a titrated or denoted dilution for an hour at room temperature or overnight at 4 °C in a moistly chamber. If a transcription factor was stained, cells were permeabilized with 0.1% Triton-X prior to blocking and incubation with the primary antibody. After washing with PBS + / + with 0.1% BSA for five minutes, cells were incubated with the secondary antibody for 30 min in a moistly chamber at room temperature, which were subsequently washed twice for five minutes in PBS + / + with 0.1% BSA. Lastly, 1:10,000 diluted 4′,6-Diamidin-2-phenylindol (DAPI) nucleus counter-staining was performed for 10 min in a moistly chamber at room temperature. Cells were mounted using Shandon™ Immu-Mount™ (Thermo Scientific, USA) onto a slide. For proving the pluripotency stage of Neural crest derived spheroids and outgrown adherent cells, following antibodies were used: Nanog 1:500 (130–117-526, Miltenyi Biotec, Germany); OCT4,1:400 ( MA5-14,845 Invitrogen, USA), Sox-2, 1:100, (53–9811-82, Invitrogen, USA), SSEA4 1:1000 (MA1-021, Invitrogen, USA), Tra-1–60, 1:500 (130–122-965 Miltenyi Biotec, Germany), Nestin, 1:2000 (130–122-965, Invitrogen, Carlsbad, USA), CD271 1:500, (130–112-794, Miltenyi Biotec, Germany). To analyze the corneal endothelial differentiation stage, antibodies against ZO-1, 1:1000, (PA5-28,869, Thermo Fisher), PITX2 1:00, (H00005308-M01, Abnova, Taiwan), Col-8, 1:500, (NBP2-30,020, Novus Biologicals, USA) and Na/ K ATPase 1:00 (MA3-924, Thermo Fisher, USA) were applicated.

### Potential Difference Measurement

The activity of the ‘Na + /K + -transporting ATPase 1α’ was analyzed as a preliminary functional verification test for corneal endothelial differentiation. The potential difference presumably established by an ATP1A1 pump activity between two compartments separated through a microporous membrane, was measured. 3000 cells per mm^2^ were seeded onto a collagen membrane with Descemet-like topography and cultivated in ESFM medium for 24 days. The differentiated cells were transferred to a ‘Transwell®’ (Corning Life Sciences, Amsterdam, Netherlands) insert. The insert was transferred to a well plate (Greiner Bio-One, Frickenhausen, Germany), in which already a copper electrode was placed beforehand in 2 ml ESFM media. Another electrode was then placed in the Transwell® insert compartment. Subsequently, 750 µL ESFM medium was added to the cells inside the insert. Both electrodes were connected to an oscilloscope to register the emerging potential difference. Concurrently, an empty Transwell® insert was analogously measured in order to subtract and hence normalize sporadically occurring potential differences. Measurement was performed until the potential difference did show a stabilized level (2.5 h). The control was seeded with a lower cell density of 5000 cells per cm^2^ on a collagen without the topography.

### Statistical Analysis

Running a two-group ANOVA gets exactly the same P value as an unpaired t-test. The given sample P-values were compared with control Anova testing was performed with Sigma Plot Version 14 (Erkrath, Germany). Values of *P = 0.05, **P = 0.01, and ***P = 0.001 were considered significant and are indicated in the figures.

## Results

### Tissue Characterization

**Fig. 1:**
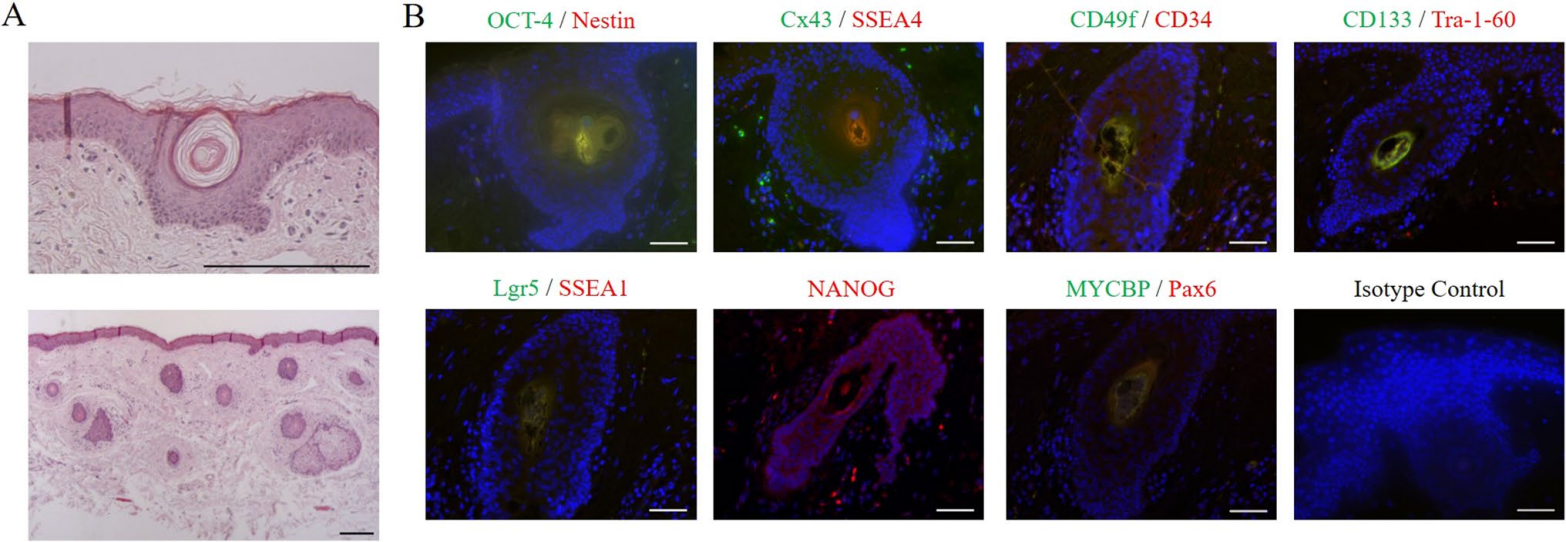
Staining of eye lid tissue section. A) Eyelid tissue sections were stained with Hematoxilin /Eosin (HE). Pictured here are hair follicle just below the epidermal skin surface. Scale bars indicate 200 µm. B) Tissue sections were stained with fluorescent labeled antibodies to indicate expression of pluripotency and neural crest associated markers within the eyelid tissue. Scale bars display 50 µm

To investigate the stem-cell potential of eyelid skin tissue samples, paraffin embedded hair follicle tissue sections were stained with hematoxylin and eosin. As demonstrated in Fig. 1A the eyelid sections displayed an abundance of hair follicles in the sub-epidermal parts of the skin tissue.

For a comprehensive characterization of the hair follicle-residing stem-cell populations, immunofluorescence analyses were performed. Since hair follicle stem-cells are commonly known to express pluripotency-associated markers, sections were stained with antibodies against Oct-4, Nang, Sox-2, SSEA4, and Tra1-60. Furthermore, other markers that are associated with neural crest cells were investigated, such as CD34, Nestin, Connexin-43, and CD49f.

As demonstrated in Fig. 1B, all measured markers were expressed. The co-localized expression of pluripotency transcription factor Oct-4 with Nestin an intermediate filament of stem or progenitor cells [23] as well as the expression of the gap junction protein Connexin-43 [24, 25] as a postulated marker for pre-migratory and migratory neural crest stem cells with the stage-specific embryonic antigen-4 (SSEA-4) a glycosphingolipid marker commonly known to be expressed in pluripotent cells which is downregulated through differentiation [26], supported the hypothesis of an immature stem cell population present in the hair follicle tissue region. This was further confirmed by the co-localized expression of CD34 with CD49f, which have been shown to be key modulators of HFSC identity in vitro [27]. CD49f is moreover involved in the regulation of stemness by the direct regulation of Oct-4 and Sox2 [28]. CD133/1 is described as a marker of developmental hair follicle [29] and together with Lgr5, a marker for cycling and long-lived hair follicle stem cells [30], the present staining could indicate the existence of cycling hair follicles in aged eyelid skin samples. Nanog, as the key regulator of stem cell plasticity and modulator of Oct-4 and Sox2, and Tra-1–60 expression did confirm the presence of a pluripotent stem cell population at the hair follicle [31, 32]. Finally, staining of Pax6 as an early neural crest marker at the hair follicle confirmed the possible presence of neural crest-derived immature stem cells in aged human eyelid skin samples [33] (Fig. 1). Taken together, hair follicle tissue from eyelid skin samples seems to be suitable for the isolation of adult stem cells with pluripotent properties.

### Cell and Sphere Cultivation

**Fig. 2:**
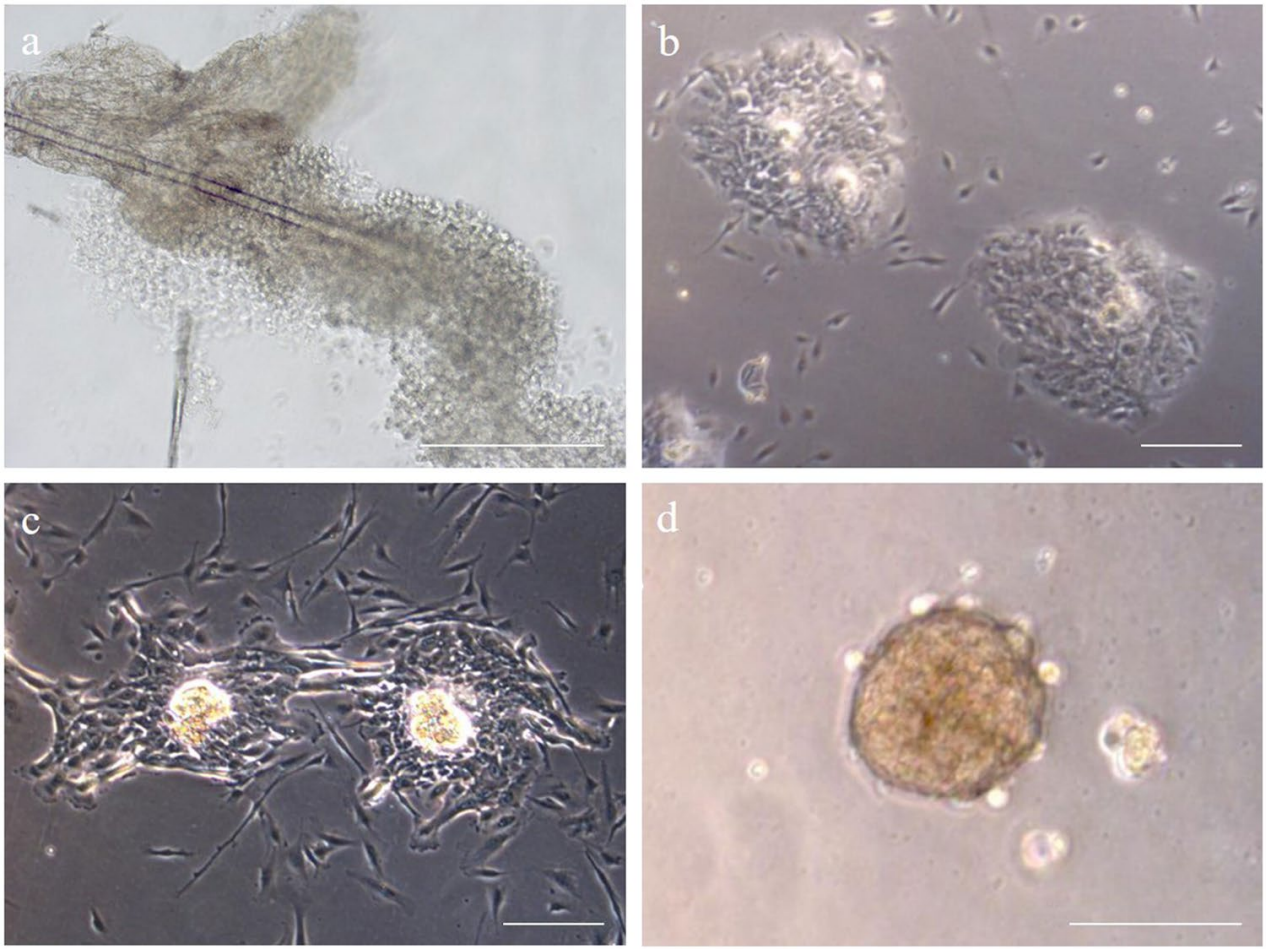
Hair follicle bulge region cells in cell culture. a) Hair follicle cells were isolated from eyelid skin and cultivated on collagen I coated tissue culture ware. b) Hair follicle stem cells arrange in circular colonies. c) In certain colonies sphere formation could be observed. d) Luminescent sphere with a diameter between 100 and 200 µm were taken for characterization or differentiation purposes. Scale bar of each picture shows 200 µm

Hair follicles isolated from eyelid skin tissue samples were applied to collagen-coated culture vessels where they started to grow out and to form colonies. Figure 2a demonstrated how cells grow out from hair follicle. We ascertained by isolating hair follicles from a lot of different donors that the outgrow duration is donor dependent and takes one to three weeks. The outgrown adherent cells displayed a low nucleus to cytoplasm ratio (Fig. 2b). Already during cell growth and spreading on the collagen coated surface, the circular arrangement of adherent HFSC were observable. In the center of cell circles, some cells began to detach from the bottom and coalesced into spheres as the cultivation time progressed (Fig. 2c). Later, the spheres began to fuse and pack together into dense cell assemblies (Fig. 2d).

**Fig. 3:**
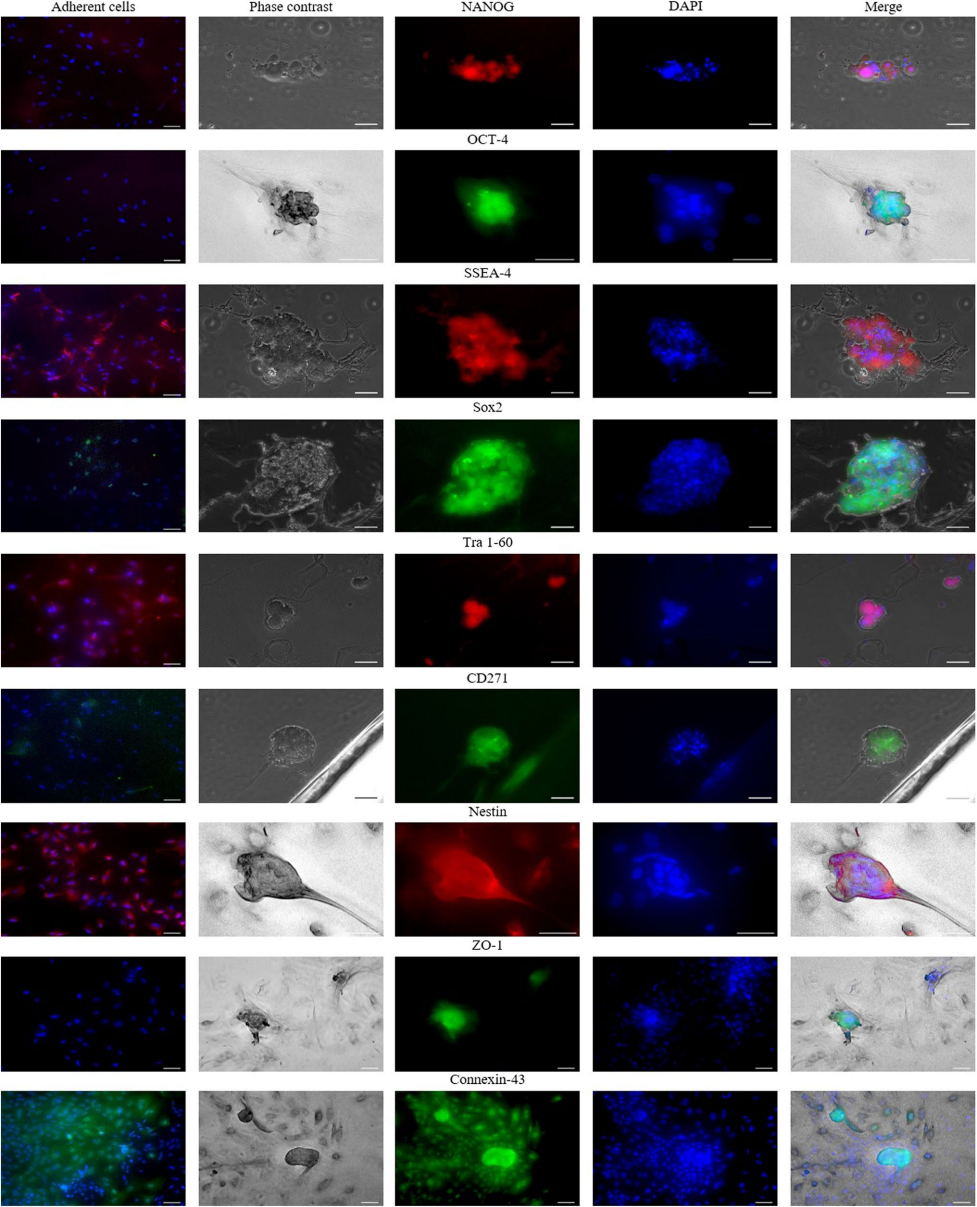
Protein expression analysis of HFSC spheres and adherent cells? Adherent HFSC and emerging spheres were stained with pluripotency and neural crest typical markers. Except for Nanog, OCT-4 and ZO-1 both adherent cells and spheres stained positively for SOX-2, SSEA-4, Tra-1–60, CD271, Nestin, ZO-1 and Connexin. Nuclei were stained with DAPI (for isotype control see supplementary data). Scale bars represent 50 µm

In order to analyze the adherent cells and the sphere population regarding their multipotent neural crest characteristics, we examined the expression of the markers Nanog, Oct-4, Sox-2, Tra-1–60, SSEA-4, Nestin, CD271 and Cx43 (Fig. 3). Spheres showed expression of all markers examined. In comparison, single outgrown cells expressed the neural crest-specific marker CD271, nestin, and connexin-43, as well as the pluripotency-associated surface proteins SSEA-4 and Tra-1–60. Following cell outgrowth on collagen, cells exhibited decreased expression of pluripotency-associated transcription factors, showing only low expression for Sox-2 and no expression at all for Nanog and Oct-4 (Fig. 3).

**Fig. 4:**
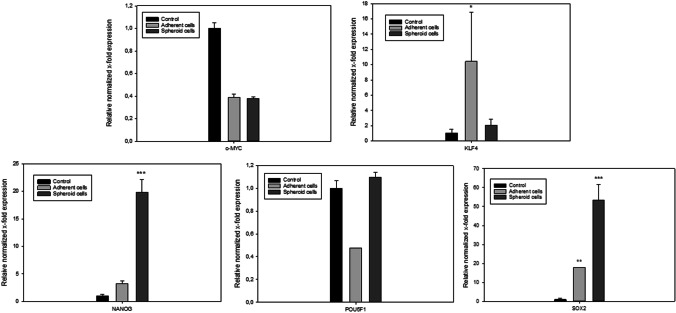
Comparison of stem cell potency associated gene expression between spheres and adherent cells. The relative normalized gene expression of Nanog, POU5F1, SOX-2 and KlF4 was examined for 3D spheres compared to single adhering cells. Gene expression data were normalized using the housekeeping genes GAPDH and B2M. Error bars represent three replicates. Significance in comparison to control was analyzed by one way anova testing. * indicates *p* < 0.05, ** indicates *p* < 0.01 and *** indicates *p* < 0.001

Furthermore, to compare the stemness of single cells and spheres, they were analyzed regarding their expression of pluripotency-associated genes *NANOG, POU5F1, SOX2, KLF4* and *cMYC* by RT –PCR analysis. (Fig. 4). The results suggest that sphere cells exhibit a phenotype with increased pluripotent properties compared to the adherent cell population. These assumptions could be confirmed by gene expression analyses. As demonstrated in Fig. 4, the sphere-derived cells showed in comparison to control cells a significant upregulation for *NANOG* and *SOX2*, whereas adherent cells expressed weaker these marker genes.

In addition, we measured the expression of the zinc finger-containing transcription factor Krüppel-like factor 4 (KLF4), which is involved in cellular processes like proliferation and differentiation [34]. Increased expression of *KLF4* in adherent cells suggests that they are more active in proliferation and maybe also in differentiation than sphere cells. In fact we gained a similar impression when observing the cell cultures as the spheres barely amplified whereas the adherent cells slowly proliferated during the first and second passage. *POU5F1* weren´t expressed wether by spheroid derived nor by adherent HF-NCSCs. Additionally, we measured the expression of *cMYC*, and were not surprised that this oncogene [35] was almost unexpressed by both adherent slowly proliferating and nearly non-proliferating sphere HFSCs. The negative result of cMyc also indicates that the measured cells do not have an oncogenic character, which is a particularly important finding for aged tissue cells [36].

### Trilineage Differentiability of NCSCs

**Fig. 5:**
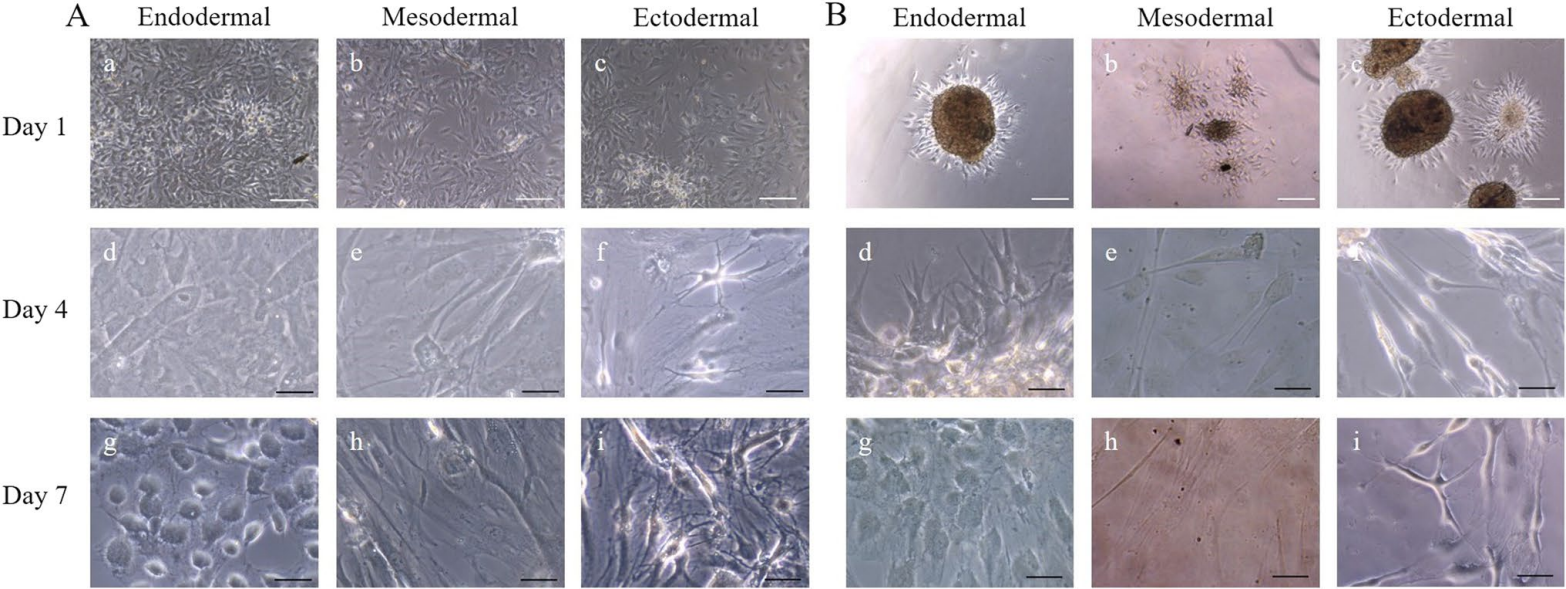
Ecto-, meso- and endodermal differentiation potential of adherent and sphere HFSC**.** Differentiation was induced with the StemMACS™ Trilineage Differentiation kit. Adherent cells (A) and sphere cell outgrowth (B) were cultivated in differentiation inducing media for six days according to the manufacturers` instructions. Starting from the third day of cultivation the cells showed an altered cell morphology. White scale bars show 200 µm and black scale bars show 50 µm

HFSC adherent cells and spheres were analyzed for their ability to differentiate along their ectodermal lineage and transdifferentiate into cells of mesodermal and endodermal phenotypic lineage using the commercial kit “StemMACS™ Trilineage Differentiation Kit”. At first as demonstrated in Fig. 5A and B morphological changes of adherent or spheres HFSCs after application of three different induction media were investigated by phase contrast microscopy. Similarly, no considerable morphological differences were detectable in adherent and sphere HFSC cultures within the first three days (data not shown). However, after four days of cultivation, cell morphological change were visible in both culture types. After 7 days, the differentiation protocol was completed and cell morphology was distinctively altered in all three cell populations for both adherent and sphere-derived HFSCs. In the course of endodermal differentiation, addition of the induction medium resulted in a roughly globular cell morphology. In addition, the differentiation process was accompanied by only a small reduction in the initially seeded cell amount. The same observations could be made for mesodermal differentiation, as many cells began to detach three days after differentiation start. Nevertheless, adherent and sphere-derived HFSCs, drastically changed their morphology under the influence of mesodermal induction and finally two distinct cell populations were visible: 1) planar and rather large cells and 2) small cells with a compact cytoplasm. Differentiation along the ectodermal lineage led to cells with a small soma and fine extensions.

**Fig. 6:**
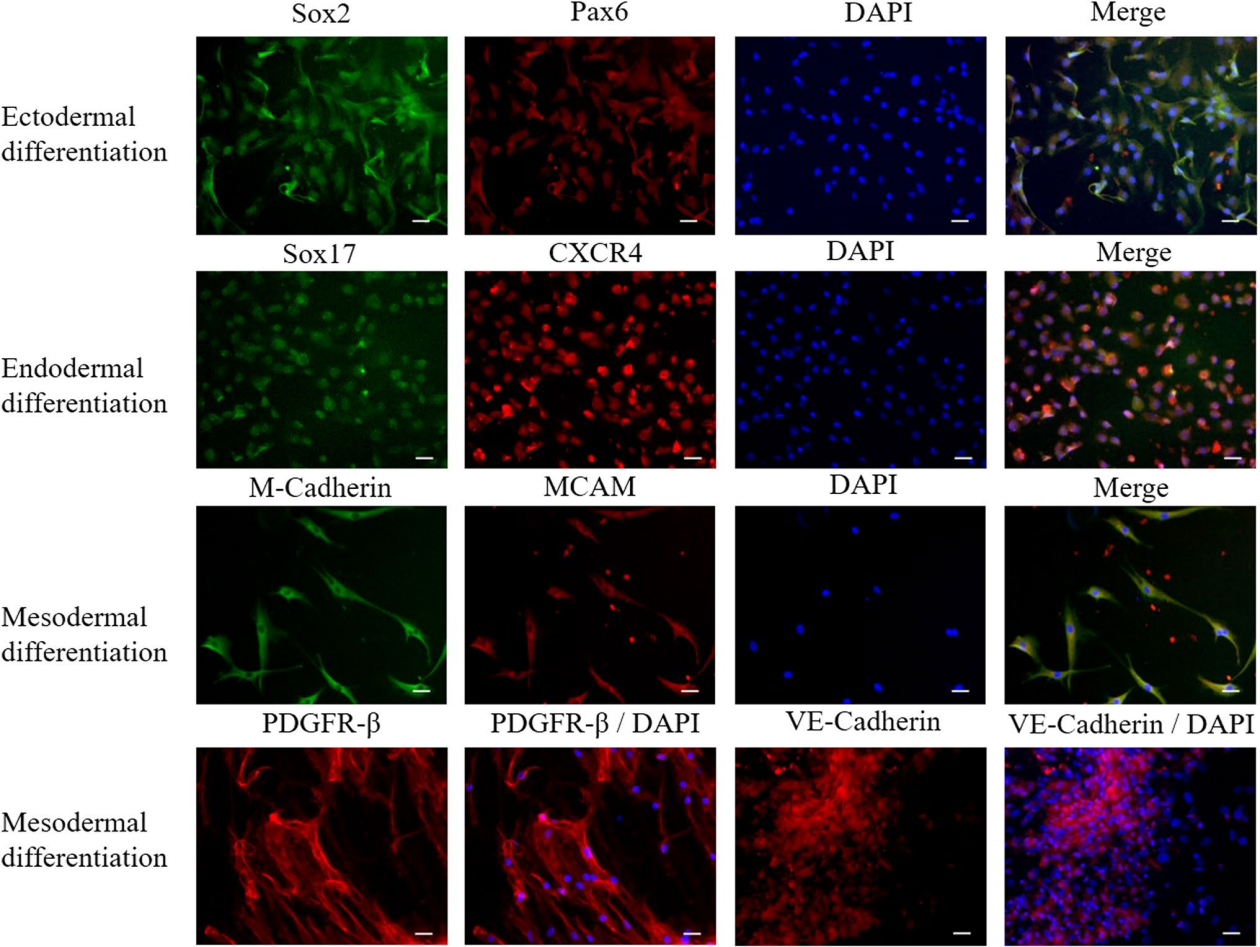
Evaluation of ecto and endo- and mesodermal differentiation of adherent HFSC by immunofluorescence. After six days of cultivation in differentiation media, cells were fixed and stained for ecto-, endo-and mesodermal specific progenitor cell markers. Exemplary results from adherent cell culture are shown. Differentiation of ectodermal progenitor cells was assessed by expression of Sox2, Pax6, Synaptophysin and NEFL. Sox17 and CXCR4 were used to identify endodermal progenitor cells. Staining of M Cadherin and MCAM as well as PDGFR β and VE-Cadherin, was performed to identify mesodermal progenitor cells. Nuclear staining was conducted with DAPI. Scale bars indicate 50 µm

To confirm differentiation, cells were stained against early lineage-specific ecto- meso- and endodermal markers. Exemplary results from adherent cell culture are shown. As demonstrated in (Fig. 6) neuro-ectodermal lineage differentiation of HFSCs revealed Sox2 and Pax6 positive cells, but due to their neuro-ectodermal origin, these markers were also present prior to the differentiation process. Therefore, we investigated the expression of the later neuronal markers Neurofilament Light Chain (NEFL) and Synaptophysin, as a definitive differentiation assessment (supplemental data). The staining patterns, although faint, were detectable in certain cells that that showed a neuronal-like phenotype. Additionally to the characteristic morphological change of the cells, the expressions of Sox2 and Pax6, indicated early and those of NEFL and Synaptophysin later differentiation into peripheral neuronal-like progenitor cells (supplemental data).

The endodermal differentiation status was confirmed by co-expression of Sox17 and CXCR4 (Fig. 6), similar to the definitive endodermal induction of embryonic stem cells before [24]. Mesodermal differentiation was hypothesized to lead to smooth muscle or pericyte progenitor-like and to vascular endothelial-like progenitor cells. Therefore, the expression of the smooth muscle cell markers PDGFR-β, M-Cadherin and MCAM and the vascular endothelial marker VE-Cadherin was examined. Both cell populations expressed either only one or the other markers, confirming the hypothesis of two cell phenotypes (Fig. 6).

In summary, the results confirmed an enhanced plasiticity of cells derived from adherent and sphere outgrown HFSCs (data not shown).

### Corneal Endothelial Cell-Like Differentiation

**Fig. 7:**
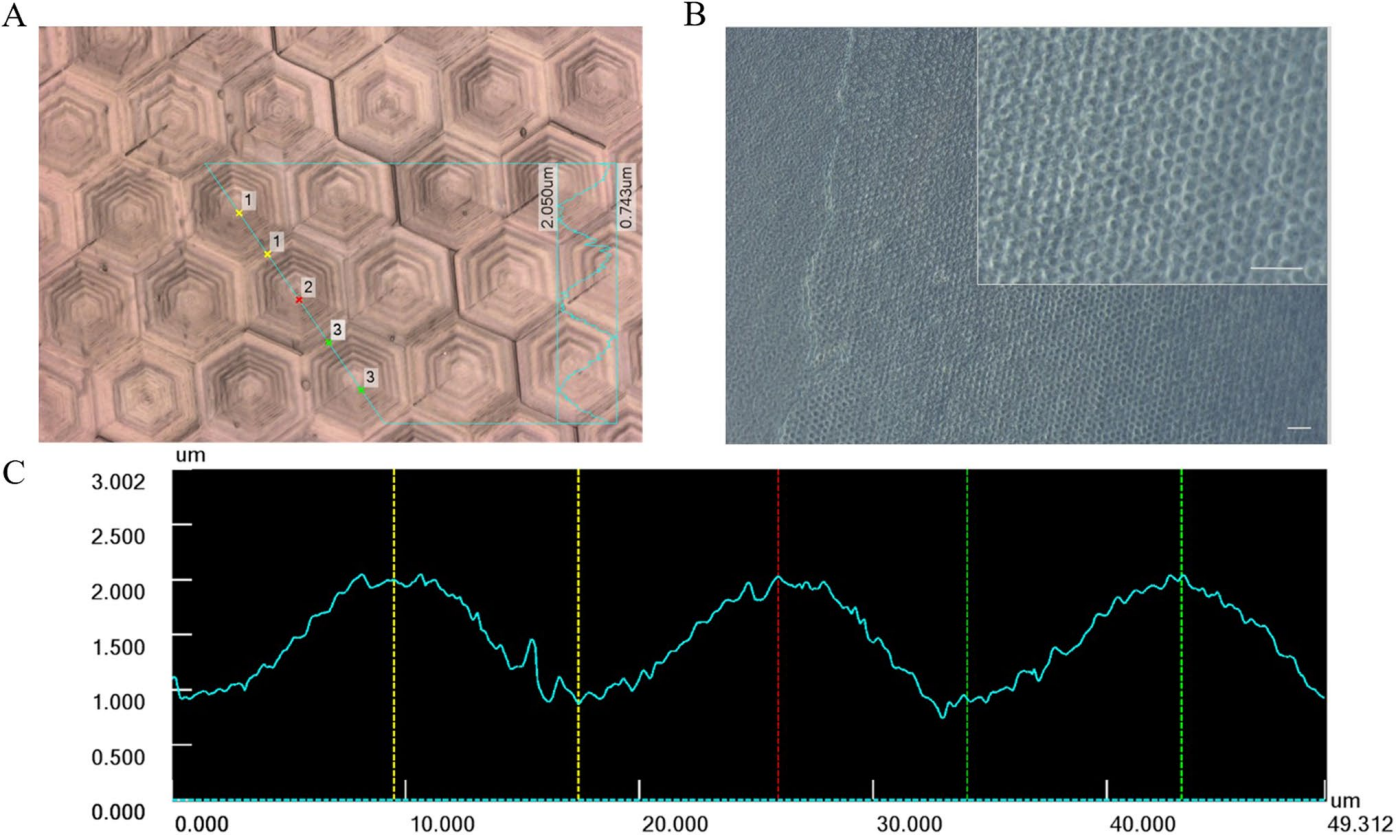
Polydimethylsiloxan "Negative" with Descemet`s microtopography was molded in collagen and used as differentiation inductor for HFSC. A) CLSM image of PDMS imprinted with Descemet like Topography (DLT) showing hexagonal elevations. B) Transfer of the DLT onto a collagen membrane. Scale bar indicates 50 µm. C) Line profile along the blue line in the CLSM image (A) that illustrates the width and height of the hexagonal elevations

Lastly, we assessed the application potential of the isolated immature HFSCs for the development of a potential corneal endothelial autograft. With Fig. 7, we want to recall the kind of biomimetic induction tool, which has been described more extensively in previous studies. Figure 7A presents a silicone imprint of the Descemet’s topography reproduced by two-photon lithography. The line profile along the blue line in the confocal laser scanning microscope (CLSM) image demonstrates the width and height of the measured hexagonal elevations (Fig. 7C).

**Fig. 8:**
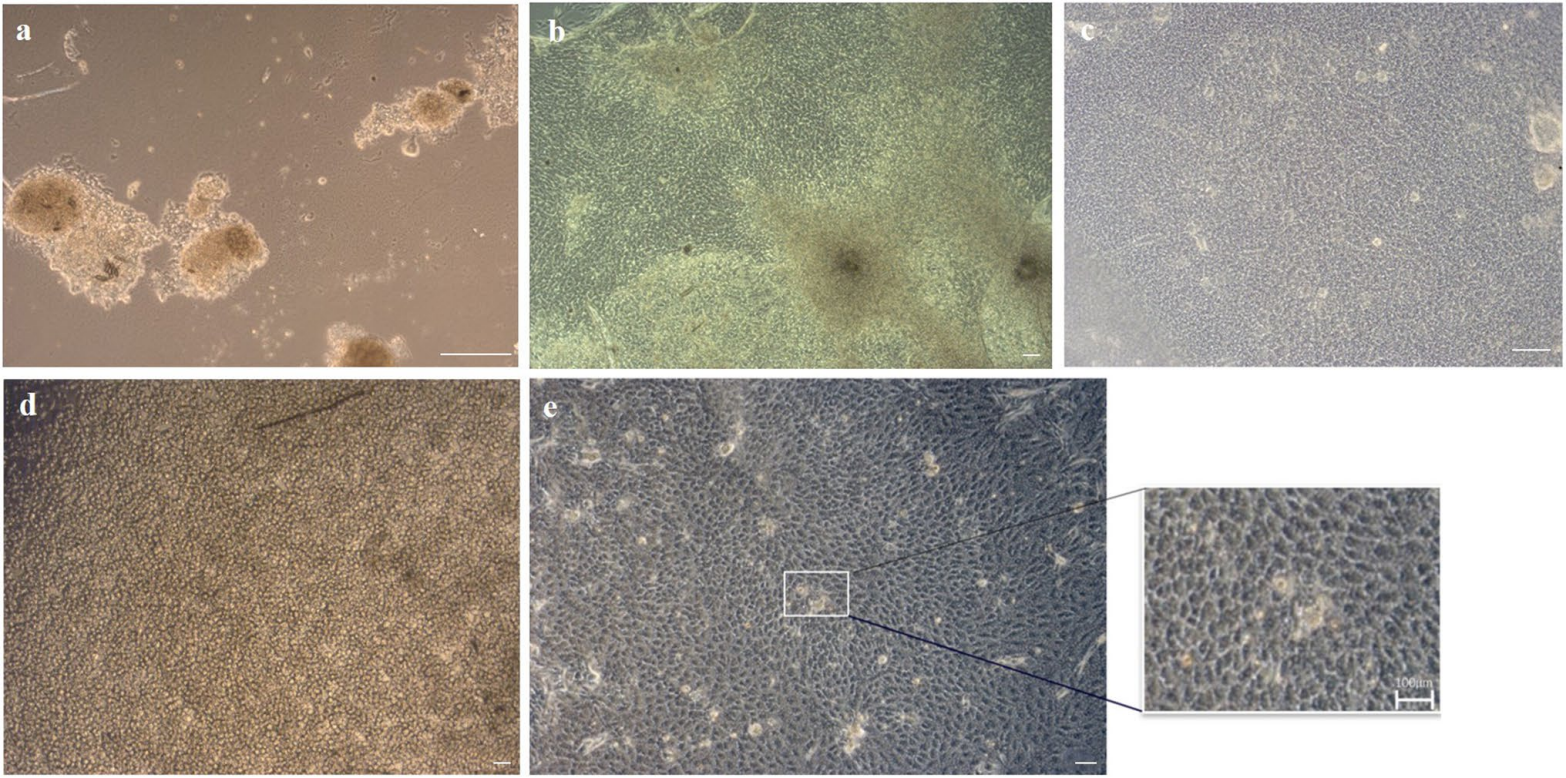
a) HFSC spheres were seeded onto the DLT imprinted collagen surface and started to outgrow after 24 h of cultivation, scale bar represents 100 µm. b) After ca. 14 days, surface was completely covered with outgrown HFSC, scale bar represents 50 µm. c) Cells within the cell layer gain a polygonal / hexagonal shape cells after ca. 20 days of cultivation, scale bar represents 50 µm. d) Adherent HFSC were seeded on DLT molded collagen at a concentration of 3000 cells/mm^2^, scale bar represents 100 µm**.** e) After 7 days of cultivation cells showed a hexagonal morphology and a small area is 4.5 fold magnificated presented. Scale bar represents 100 µm

As demonstrated in Fig. 8a, spheres with a diameter between 100 and 200 µm were seeded onto a DLT-imprinted collagen surface. Spheres attached to the surface and first single cells migrated out of the spheres after 24 h of cultivation. The outgrown cells initially displayed a mixed morphology, most were polygonal, some were round and a few showed a stretched shape (Fig. 8b). During the cultivation process, the outgrown cells of neighboring spheres reached each other and stopped their proliferation and spreading due to contact inhibition by cell–cell contacts to neighboring cells. 20 days after confluence was reached, the morphology of the adherent cells changed more and more into a polygonal or hexagonal cell form (Fig. 8c). For comparison, we also seeded adherent cells of passage 1 onto the DLT-imprinted collagen surface. After only 24 h, we observed an even distribution of cells on the membrane. (Fig. 8d). Figure 8e demonstrates the layer of hexagonal / polygonal cells after 7 days of cultivation.

**Fig. 9:**
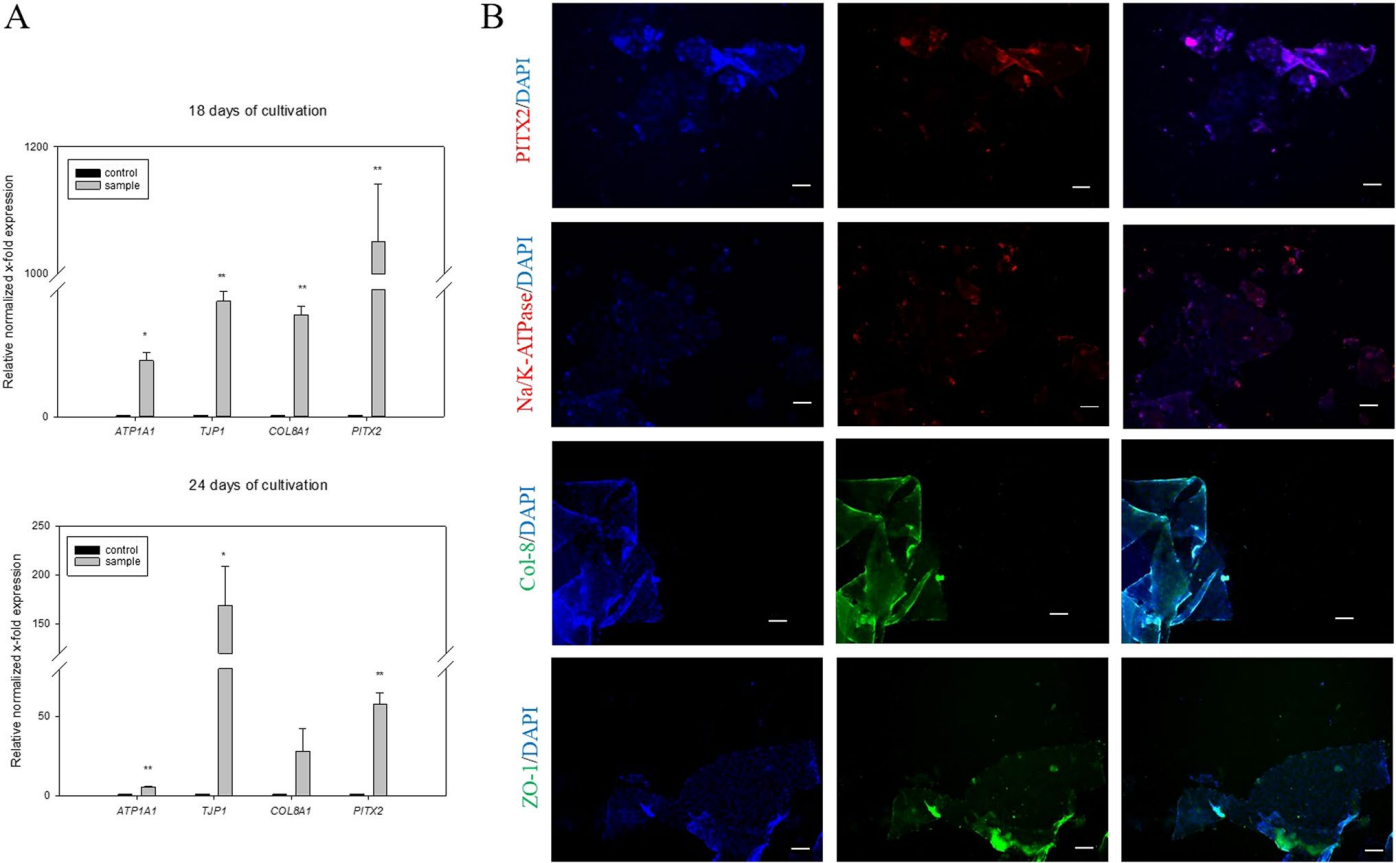
Corneal endothelial cell associated gene expression of differentiated adherent and sphere HFSC. Analysis of the relatively normalized gene expression of ATPase, ZO-1, COL8 and PITX2.A) Adherent HFSC of passage 1 were seeded on a DLT imprinted collagen in a density of 3000 cells/ mm^2^ and cultivated for seven days. Gene expression data were normalized using the housekeeping genes GAPDH and B2M. Error bars represent three replicates. Relative gene upregulation was assessed in comparison to Jurkat cells as control cultured on tissue culture plates in a seeding density of 1000 c/cm^2^. B) Sphere outgrowth HFSC were seeded on a collagen membrane with DLT and cultivated for 21 days. Relative gene upregulation was assessed in comparison to Jurkat cells as control cultured on tissue culture plates in a seeding density of 1000 c/cm^2^. Significance in comparison to control was analyzed for A) and B) by one way anova testing. * indicates *p* < 0.05, ** indicates *p* < 0.01 and *** indicates *p* < 0.001. C) Immunofluorescence analysis was performed to measure marker expression of PITX, Na/K-ATPase, Col-8 and ZO-1. Sphere grown cells were cultivated on DLT collagen for 14 days. For antibody staining single cells were scraped off the collagen I scaffold. Nuclei were stained with DAPI. Scale bars show 50 µm

To verify that adhesion of HF NCSCs on the biomimetic DLT substrate induce differentiation, expression of corneal endothelial-specific markers *PITX2*, *ATP1A1*, *Col8A1* and *TJP-1* were analyzed at the gene level by RT-PCR. Gene expression analyses were performed 18 and 24 days after seeding for adherent and sphere-outgrown HFSCs, respectively (Fig. 9). For sphere-outgrown cells, significantly enhanced gene expression was measured for *PITX*, *ATP1A1* and *TJP1* compared to control cells (Fig. 9B) as *PITX2* was upregulated 57.98-fold, *ATP1A1* 5.76-fold, and *TJP1* 168.96-fold. By virtue of the high standard deviation the gene expression of *COL8A1* with 28.26-fold enhanced expression is not significant. Adherent cells seedings revealed a significant expression level enhancement for each measured gene. In comparison to Jurkat cells (Jurkats also express Na /K ATPase), *ATP1A1* was upregulated 44.0-fold, and *TJP1* was 90,91-fold enhanced. An 80.34-fold upregulation of *COL8A1* was measured and *PITX2* even reached a 1051.28-fold gene expression. Basically, it has to take into account that independent of the cell seeding strategy, the optimal time point for RT PCR measurement is uncertain. For this publication we demonstrated one of the exceptions, by which we measured gene expression enhancement for all genes to the same point of time. As documented in our previous publication, the gene expression of individual genes starts to different times and we got therefore also often data where we couldn´t measure all genes to the given time point but instead only three or two. (data not shown). In contrast to RT PCR for generating appropriate data from protein expression analyses we always started the immunofluorescence measurements one week after proper corneal endothelial morphology of the samples were established.

In most analyses, protein expression was measurable but only unsatisfactorily, since the collagen substrate soaked up the antibody-enriched liquids during the staining procedure and it was impossible to wash out the nonspecifically bounded antibodies. As a consequence, the high background fluorescence of the collagen membrane disturbed an appropriate assessment of the marker expression. However, pieces of self-organized cell layers that detached from the underlying collagen membrane through fixation procedure were positive stainable for all measured corneal endothelial markers. (Fig. 9C). As required, the staining of the transcription factor PITX is located in the nucleus. Na^+^/K^+^-ATPase is visible as red pits at the cell boundaries. Col8 as an extracellular basement matrix protein is randomly distributed and ZO-1 as a tight junction-associated protein is rather weakly but marginally detectable at the cell boundaries.

### Functional Assay

**Fig. 10:**
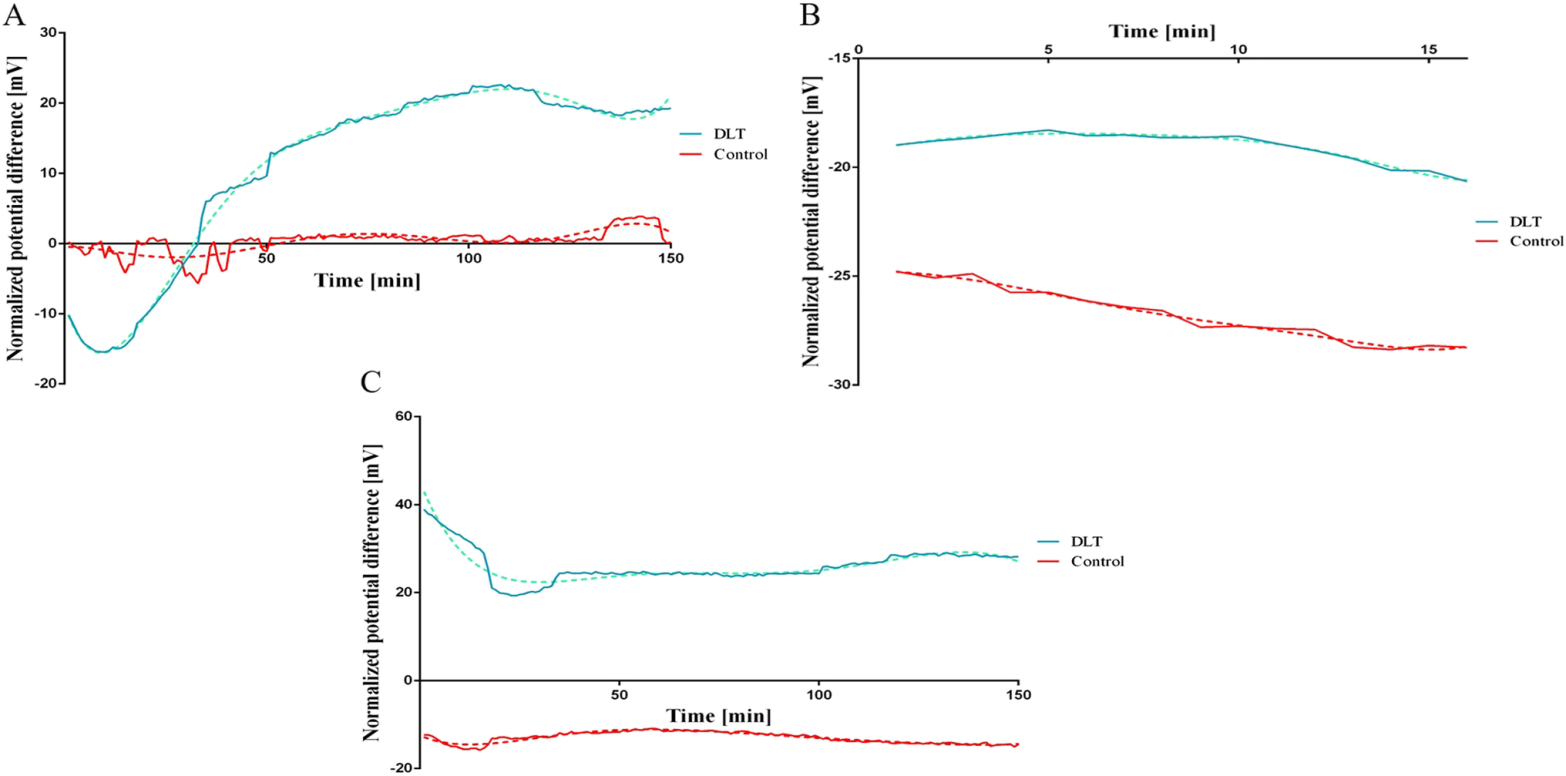
Functional analysis of differentiated HFSC by potential difference. A) Collagen membranes seeded with HFSC for 24 days were transferred into a Transwell® cultivation system with a membrane porous for sodium and potassium ions. The potential difference between the upper and lower compartment was measured and normalized to a reference measurement consisting of only a Transwell® insert. Green line indicates a collagen membrane seeded with 3000 cells per mm^2^ on a Descemet’s like topography (DLT), while red line indicates a collagen membrane seeded with 5000 per mm^2^ cells on an unstructured control collagen. Dashed lines indicate nonlinear sixth order polynomial regression fit curves. The mean value of 540 measurements in each minute is displayed. B) Potential difference between DLT and control collagen while flushing with distilled water. After the first measurement as displayed in (A), collagen membranes were incubated at 37 °C for 12 h to reach a definite equilibrium between the two compartments. After incubation cells were flushed with distilled water for 16 min and 40 s. An outward ion movement into the lower compartment results in a negative potential difference. DLT cultivated cells on collagen caused a decreased ion movement, which resulted in a lesser negative potential. C) Potential difference measurement after of 12 h of incubation and flushing. After flushing the distilled water was exchanged for differentiation medium and the potential difference was measured again for 150 min. The potential difference for cells cultivated on a DLT structured collagen membrane started at + 40 mV, but stabilized eventually after an initial decline at approximately + 25 mV after 30 min incubation. The control collagen remained stable as previously observed in the first measurement (confer to Fig. A), but the value fluctuated around -10 to -15 mV

One of the key mediators of corneal endothelial functionality is a pumping Na–K-ATPase activity. For a potential future application as an autograft consisting of corneal-endothelial like cells derived from NCSCs, these grafts should not only express corneal endothelial markers, but also more importantly show CE functionality. As a proof of concept experiment, we measured the electrical potential difference across to compartments in a newly established experiment setup. If an enzymatic activity is present from the differentiated cells, then a potential difference should be measureable due to an ion gradient established by the Na–K-ATPase. Twelve well plates with transwell inserts were each equipped with two electrodes to record the voltage differences between the apical and basal compartments. The readings were recorded every 60 s over a period of 150 min at a frequency of 10 values per second. The voltage difference between a collagen membrane carrying a layer of differentiated corneal endothelial like cells (CECs) and a cell-free collagen as control was measured. The membrane potential of the HFSC-loaded collagen was initially 10 mV lower than the control collagen (Fig. 10A). Within the next 32 min, the potential constantly rose to the baseline (control level) and beyond until a plateau value of about + 20 mV was reached after approximately one hour of measurement. The curve shows that the cells were able to establish a potential difference of 30 mV and maintain a potential of approximately 20 mV. In a subsequent experiment, the capacity of HFSC was investigated concerning their ability to re-establish a potential difference. In order to destroy the existing equilibrium (potential difference of 20 mV), the membranes or rather both compartments were rinsed with distilled water. And in fact, as demonstrated in Fig. 10B, the potential immediately dropped into the negative range after rinsing. After addition of medium, the potential difference built up again until it reached + 40 mV. After 30 min it stabilized at 20 mV, which was approximately 30 times higher than the control membrane without cells (Fig. 10C).

In summary, the evidence that the cells were able to actively establish a potential difference between two compartments is another indication of successful differentiation.

## Discussion

Corneal dystrophies are one of the main causes of corneal opacities and present a major challenge for modern medicine. Current treatments such as the DSAEK are based on the transplantation of donor tissue. However, due to the severe shortage of transplants, many patients cannot be helped [13].

Not surprisingly, urgent attempts have been made for decades to circumvent the problem of corneal shortage by either attempting to multiply adult CECs in vitro [37–39] or to differentiate stem cells into corneal endothelial-like cells [40–43]. Our approach is to develop a coherent functional corneal endothelial layer from stem cells of adult origin which has not been reprogrammed or induced by use of biochemical pleiotropic factors. In this context, we have recently successfully differentiated stem cells from foreskin [17, 22] and Wharton’s Jelly tissue into corneal endothelial-like cells by cultivation on a biomimetic substrate that simulates the environment of the Descemet's membrane. With this study, we aimed to demonstrate that stem-cell source can be differentiated, which in principle allows the production of autologous corneal endothelial tissue for any age group. Considering that the mean age of patients suffering from corneal endothelial dystrophy is approximately 60 years [44], we intended to use stem-cell reserves from patients with a similar age between 53.5 ± 7.1 years. In contrast to young stem cells, stem cells of older tissues do not have a nearly endless functional capacity [45]. Over the course of a lifetime, stem cells become increasingly stressed to maintain tissue integrity, which is associated with a decline in stem-cell activity [46]. In addition to the inherent self-regenerative capabilities of stem cells, the stem-cell niche is a major key modulator with respect to the prolongation of their functionality. It is fascinating that the plasticity of inherent cells of the remaining hair follicle is still preserved [43, 44], although an age-related decrease of hair follicle stem-cell niches take place. Eyelid hair follicle cells, like CECs, originate from the cranial neural crest and migrate into facial areas such as the cornea or eyelid during the seventh week of embryonic development [47]. Since we were able to obtain stem cells with high plasticity and a corresponding developmental origin from eyelid biopsies of elderly patients, we were motivated to further investigate the plasticity of HFSCs and their potential to differentiate into CECs. One of the biggest challenges was to isolate the cells with the highest plasticity from the hair follicles.

Studies in young mice demonstrated that the follicle bulge region of whiskers contains keratinocyte stem cells as well as stem cells of the melanogenic lineage and pluripotent neural crest stem cells (NCSCs) [48]. Furthermore, these studies confirmed that the pluripotent NCSC population of the hair bulge possesses high intrinsic plasticity and gives rise to an entire array of cranial neural crest-derived cell lineages [49]. Staining of eyelid tissue sections, revealed the presence of pluripotency associated markers (OCT-4, NANOG, Tra-1–60, SSEA-4 and SSEA-1), as well as the neural crest-defining markers (Pax6, CD34, CD49f, Nestin, Connexin, and Lgr5) in the follicle bulge region. In addition, it was possible to isolate and cultivate proliferating cells form the hair bulge region of aged eyelid tissue without the addition of mitogens like serum proteins. However, in contrast to HFSCs from young mouse whiskers, those from aged sample tissues lost their proliferative capacity and typical morphology after only two passages (data not shown). Nevertheless, the cells of the first passages were proliferative enough to colonize the entire cell culture plate. Moreover, some cells seemed to have an increased stem-cell potential as they were able to spontaneously form spheres. Sphere cultivation has often been shown to be more advantageous than two-dimensional adherent cell culture. Not only does the cultivation system favor the maintenance of intrinsic phenotypic properties of stem cells, but also the enhanced expression of stemness-associated markers. This might be due to the fact that the physicochemical environment is similar to that experienced by cells in vivo, for example, through hypoxia [50, 51]. Comparison of gene expression levels of *NANOG*, *POU5F1* and *SOX2* confirmed a stronger expression of these genes in sphere-forming cells compared to adherent cells. However, the weak expression of Oct-4 in all measured samples was remarkable. Immunofluorescence analysis confirmed that the adherent cells were of lower potency than sphere-forming cells, as they were not stainable against Nanog or Oct-4 antibodies. Given the recent demonstration of the importance of Oct-4 for the self-renewal capacity of HFSCs, a correlation of the poor proliferative capacity of aged stem cells and their low *POU5F1* expression seems reasonable. Similarly, embryonically-derived NCSCs exhibited loss of their stem-cell potential after two days of cultivation. This observation was interpreted as the result of stochastic signals exchanged between densely maintained cells, which spontaneously differentiated into melanocytes or into alpha smooth muscle actin-positive cells [52–54]. Even though we have not investigated in which directions the cells differentiated, we hypothesize that *POU5F1* was downregulated through ongoing cell differentiation. Since the expression of NANOG depends on the cooperative induction of *POU5F1* and *SOX2*, it is not surprising that it could not be detected at the protein level.

However, both adherent cells and spheres showed the expression of other pluripotency-associated markers, such as Sox-2, Tra-1 60, and SSEA-4, as well as all investigated neural crest-associated markers, including Nestin, Cx-43 and CD271.

Considering their lingering stem-cell potential, we examined HFSCs for their capacity to differentiate into cell lineages of the meso-, endo-, and ectodermal germ layers. Phenotypic changes of stem cells isolated from tissue samples of elderly donors underlined their tri-lineage differentiation capacities. Furthermore, the hypothesis that eyelid tissue might be a source of pluripotent stem cells was confirmed.

Based on the evidence of their high plasticity, we investigated the ability of HFSCs to differentiate into corneal endothelial-like cells.

Considering that both eye-lid-derived HF NCSC and CECs are of ectodermal origin, HFSCs containing NCSCs to an undetermined degree should prove to be a suitable cell source for CE autograft. And indeed, HFSC spheres grew out on the DLT-structured collagen surface and adopted a polygonal-like morphology. This observation led to the hypothesis that the biomimetic substrate may have induced changes in cellular and nuclear morphology and subsequently differentiation via mechanotransductive signaling pathways. Since numerous spheres were seeded, excessive cell outgrowth and overgrowth was prevented by removing the spheres as soon as the cells that grew out in a circular pattern became attached to each other. While the cells had a rather undefined roundish morphology immediately after outgrowth, they adopted a more characteristic polygonal, sometimes even hexagonal shape as the cultivation period progressed. For approving if corneal endothelial differentiation were taken place, we analyzed the gene expression of the corneal endothelial cell-typical markers ZO-1, Na K ATPase, PITX, and Col-8. The resulting gene expression levels were comparable to the results obtained by Inagaki et al. [21], who worked with eyelid skin-derived progenitor cells. Both the choice of cells and the final objective of their study were comparable to our approaches. However, they used a quite different underlying procedure than we did, as they biochemically differentiated dermal skin-derived progenitor cells and worked with FCS-containing media. Upon addition of FCS, the cells adopted a fibroblast-like morphology, which was accompanied by reduced plasticity and might possibly the reason why cells were no longer able to recognize the DLT topography. Similar observations were made by Mignone et al., as they demonstrated differentiation into smooth muscle actin-positive cells in the presence of FBS [55]. Considering that corneal endothelial cells are derived from the neuroectoderm, we tried to prevent the cells from differentiating into mesodermal lineages from the outset and instead followed culture protocols of groups that intended to differentiate HF-NCSC into neuronal lineages [56]. Among other things, we added EGF and FGF to the basal medium because these molecules, normally provided by feeder cells [57], play an important role in maintaining the HF-NCSC phenotype. Our observations revealed that the morphology of aged HF-NCSC was still preserved in passage one. Without the use of FCS, we were unable to expand HF-NCSCs and obtain cell amounts that would have allowed comprehensive flow cytometric measurements, we therefore only measured the stem cells positive for CD271, Nanog and Sox-2 (supplementary data). However, the cell amount was sufficient for characterization by immunofluorescence and for corneal endothelial differentiation.

A closer look at the gene expression data, however, revealed that the expression level of CEC-typical markers, based on HFSC sphere outgrown cells, was rather weak compared to our data from previous studies. In addition, the progression of sphere outgrowth varied with each donor. Only one out of ten sphere seedings was successful because the spheres either failed to attach or the outgrown cells exhibited an insufficient proliferative capacity and were unable to colonize the entire DLT surface. Therefore, we changed our procedure and seeded 3000 adherent single cells from passage 0 or passage 1 per square millimeter onto the DLT surface and measured gene expression then when we observed a proper corneal endothelial typical morphology. The resulting gene expressions increased up to 25-fold and were consistent with our previous data. We were surprised that single cells, which exhibited a lower potency in the first part of our study and were trypsinized before seeding, gave better results than cells grown directly from spheres. However, we assume that the plasticity of adherent single cells was sufficient because differentiation did not require a germ-layer change and, thus, no transdifferentiating event. Regardless of the seeding procedure, measurement of CEC-marker protein expression was nearly impossible. The generated CE-like layer was strongly adherent to the collagen I scaffold, and the morphology could only be clearly visualized by phase contrast imaging because the collagen substrate is not only autofluorescent, but also absorbs fluorescent antibodies, which could not be washed out. Nevertheless, in agreement with von den Bogerd et al. [58], we believe that both their hexagonal morphology and the expression of tissue-specific markers indicate that the cells exhibited a corneal endothelium-like character.

After some cells were detached from the developed layer, the corneal endothelium-associated antigens could be detected by immunofluorescence analysis. Especially PITX2 is a key transcription factor that has been detected in nuclei and is involved in the signal transduction of the migratory neural crest derived stem-cell fraction to the corneal endothelium in the embryogenesis [24]. One of the most important markers for CEC characterization is the tight junction protein ZO-1 [59]. Although single adherent HF-NCSCs were unable to express ZO-1, it could be detected in DLT-induced cells. However, following cell detachment from the cell layer, the protein was only marginally detectable at the cell borders. Expression of the basement membrane-typical extracellular matrix protein Col-8 was extensively detectable. Therefore, we assumed that the cells started to generate their own Descemet’s-like matrix. As expected, the expression of Na K ATPase was detected as spots at the cell membrane borders. The functionality of Na K ATPase was indirectly determined when the cellular pumping power was measured. This ion exchange transporter is primarily responsible for maintaining the water content of the overlying stromal layer in equilibrium and, thus, ensuring corneal transparency. The differentiated HF-NCSCs were able to establish and maintain a potential difference of approximately 30 mV.

Taken together, we identified eyelid epidermal skin as a promising cell source for hair follicle-derived NCSC to generate corneal endothelial autograft. We demonstrated that sphere-derived and DLT-induced HF-NCSC were able to differentiate into CEC-like cells. They exhibited a CEC-characteristic morphology, expressed corresponding markers and showed functional properties. Nevertheless, it should be considered that the method of sphere outgrowth is not applicable to future clinical concerns. Since we intended to start the differentiation process with the most potent and pristine cells, we considered this method to be the most promising approach. Later on in the study, we switched to single cell seeding and obtained excellent cell morphologies and gene expression data. Even after the second passage, which may already be associated with senescence in cells from older donors, HF-NCSCs still exhibited plastic properties. In future studies, we therefore intend to work exclusively with single HF-NCSC and to prove differentiation not only by four markers, but to perform comprehensive characterization to further approach clinical applicability.

## Conclusion

In summary, we have demonstrated that an immature neural crest stem-cell population can be isolated from human hair follicles of eyelid skin samples from elderly patients. These cells were able to (trans-) differentiate into cells from all three germ layers. Furthermore, we showed that HFSC could differentiate directly into corneal endothelial-like cells, which were able to maintain an electrochemical potential difference that was likely established by sodium/potassium ATPase activity. Therefore, due to their autologous origin and lack of tumorigenicity, these eye lid derived HFSCs may be a promising cell source for corneal tissue engineered grafts in the near future.

## Supplementary Information

Below is the link to the electronic supplementary material.

Supplementary file1 (JPG 52 KB)

Supplementary file2 (JPG 63 KB)

Supplementary file3 (JPG 80 KB)

Supplementary file4 (JPG 40 KB)

## Data Availability

All authors make sure, that all data and materials support the published claims and comply with field standards.
