# Supplementary figures and images for: Mechanotransductive Differentiation of Hair Follicle Stem Cells Derived from Aged Eyelid Skin into Corneal Endothelial-Like Cells
Source: Stem Cell Rev Rep. 2021 Sep 13;18(5):1668–85. doi: 10.1007/s12015-021-10249-0 (PMC9209348; doi:10.1007/s12015-021-10249-0)

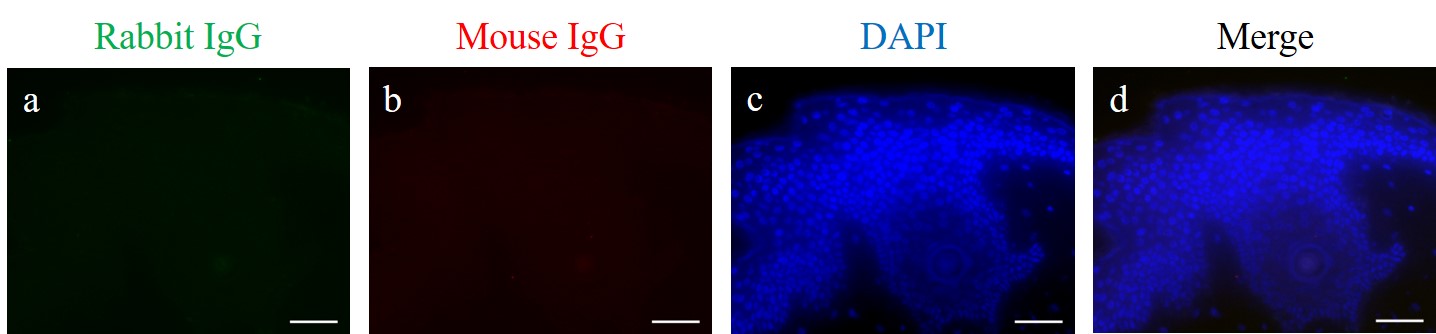

Supplement: Supplementary file 1 — Supplementary file1 (JPG 52 KB) [file 12015_2021_10249_MOESM1_ESM.jpg]

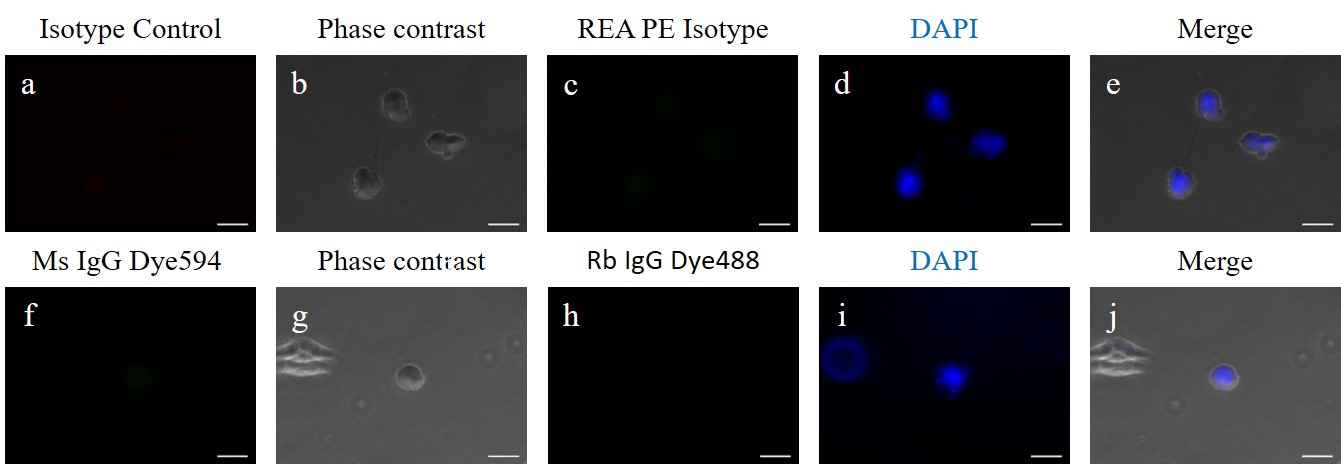

Supplement: Supplementary file 2 — Supplementary file2 (JPG 63 KB) [file 12015_2021_10249_MOESM2_ESM.jpg]

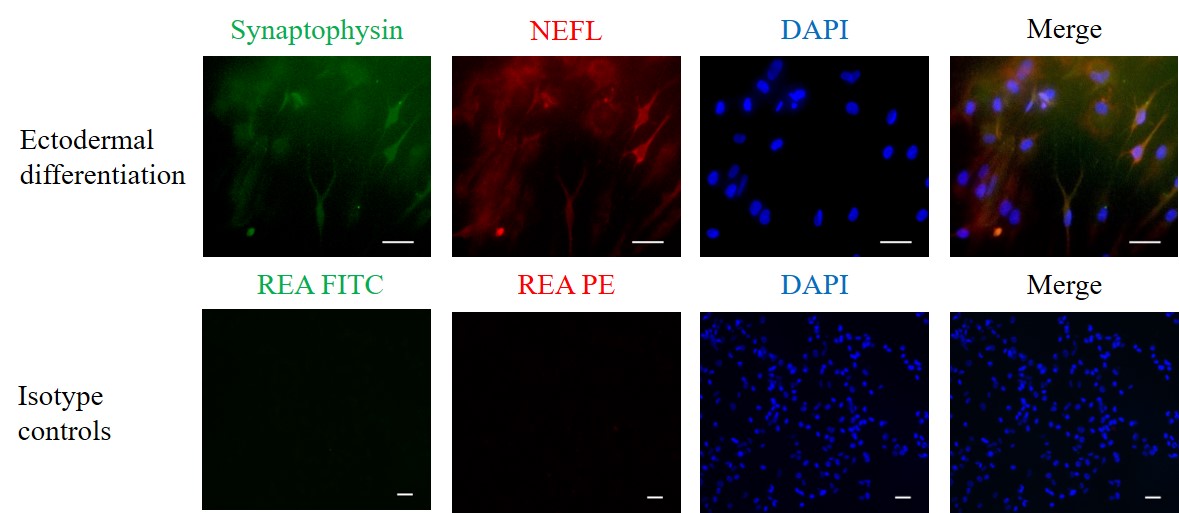

Supplement: Supplementary file 3 — Supplementary file3 (JPG 80 KB) [file 12015_2021_10249_MOESM3_ESM.jpg]

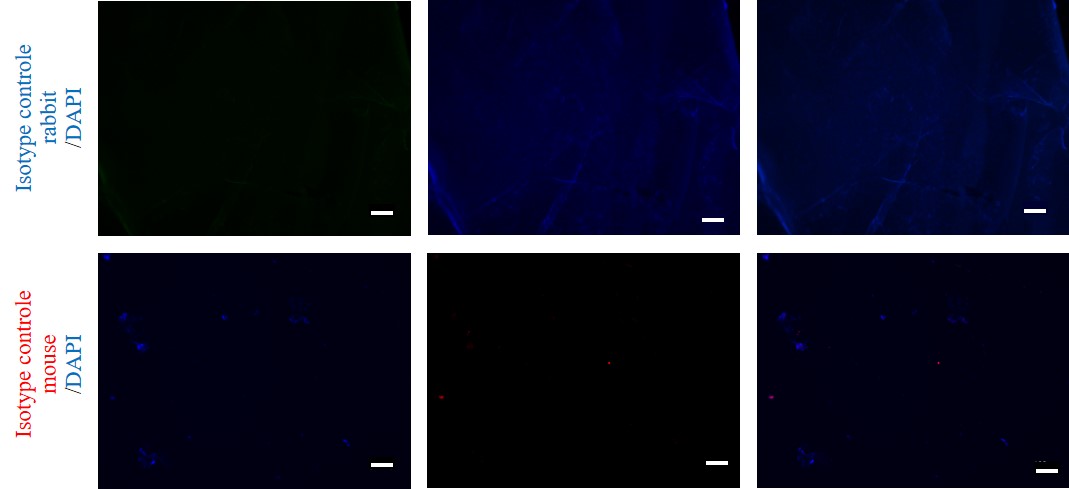

Supplement: Supplementary file 4 — Supplementary file4 (JPG 40 KB) [file 12015_2021_10249_MOESM4_ESM.jpg]
